# Successful defibrillation using double sequence defibrillation: Case reports

**DOI:** 10.1097/MD.0000000000024992

**Published:** 2021-03-12

**Authors:** Hyo Jeong Choi, Hyun Noh

**Affiliations:** a Department of Emergency Medical Technology, Sun Moon University. 70, Asan-si, Chungcheongnam-do; b Department of Emergency Medicine, College of Medicine, Soonchunhyang University Bucheon Hospital, 170, Jomaru-ro, Bucheon-si, Gyeonggi-do, KR.

**Keywords:** cardiopulmonary resuscitation (CPR), defibrillation, double Sequential Defibrillation, refractory ventricular fibrillation

## Abstract

**Introduction:**

Defibrillation is effective and the most common treatment for ventricular fibrillation (VF) and pulseless ventricular tachycardia in patients with cardiac arrest.

**Patient concerns:**

Recently we experienced 3 cases refractory ventricular fibrillation (RVF) which was successfully terminated with double sequence defibrillation (DSD) in our emergency department, so we’d like to report and discuss it.

**Diagnosis:**

Cardiac arrest

**Interventions:**

A single defibrillation 200J was performed twice for patients with ventricular fibrillation in the initial rhythm of the emergency room. At the same time, intubation and intravenous access were achieved and epinephrine and amiodarone were administered. The 400J DSD was performed on RVF patients with sustained VFs, despite several trials of 150-200J defibrillation and adherence to advanced cardiac life support.

**Outcomes:**

All three RVF patients recovered spontaneous circulation after DSD.

**Conclusion:**

The three cases we have shown are small, but DSD improves the chance of spontaneous circulation. Therefore it is suggested that attempts of DSD to patients with RVF, especially in the prehospital stages as a way to improve the return of spontaneous circulation.

## 1 Introduction

Defibrillation is effective and the most common treatment for ventricular fibrillation (VF) and pulseless ventricular tachycardia in patients with cardiac arrest.^[1–3]^ Refractory ventricular fibrillation (RVF) is defined as VF that is resistant to at least three defibrillation attempts, 300 mg of amiodarone, and does not exhibit return of spontaneous circulation (ROSC) after 10 minutes of cardiopulmonary resuscitation (CPR).^[4]^ In this situation, the application of Double Sequential Defibrillation (DSD) can be considered.^[5]^ Recently we experienced 3 cases RVF which was successfully terminated with DSD in our emergency department (ED), so we’d like to report and discuss it. IRB (Soonchunhyang University Bucheon Hospital Institutional Review Board) has exempt the patient's consent for publication. IRB is organized and operates accordiong to ICH-GCP and applicable laws and regulations. (No. of IRB SCHBC_IRB_2018-12-015)

## 2 Case report

### 2.1 Case 1

**Figure 1:**
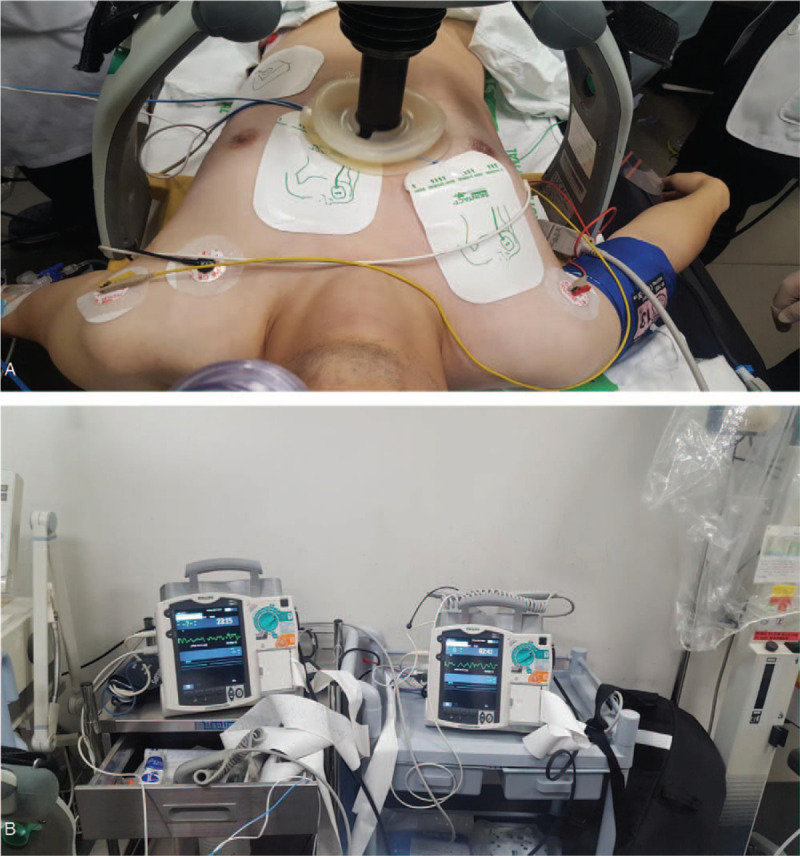
A. Apply a pad to the patient for Double Sequence Defibrillation. B. Two Defibrillators prepared for Double Sequence Defibrillation.

**Figure 2:**
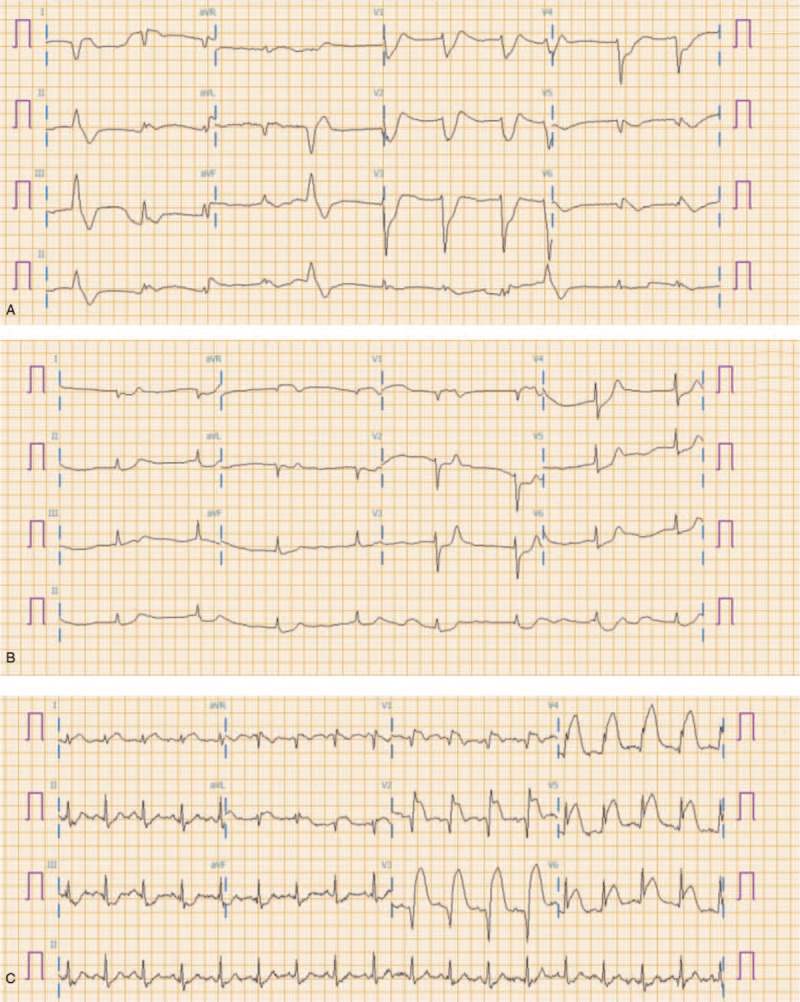
Electrocardiogram after Return of Spontaneous Circulation (ROSC) A. CASE1 B. CASE2 C. CASE3.

A 31-year-old man without any medical history was transported to the ED by ambulance due to breathing difficulties and drowsy mentality. No bystander CPR was performed because the dispatcher did not recognize the cardiac arrest. Upon arrival at the scene, the paramedic recognized the cardiac arrest and performed chest compressions and attached the automated external defibrillator. The initial electrocardiogram (ECG) rhythm at the scene was VF, and a total of 6 defibrillations was performed with the energy of 150 joules (J) until arriving at the ED but the spontaneous circulation wasn’t recovered. The initial rhythm at the ED was VF, so 200J of defibrillation was performed twice. At the same time, intubation and venous access were achieved and epinephrine and amiodarone were administered. ROSC was achieved 7 minutes after arrival at the ED but wasn’t sustained. 3 minutes after ROSC, VF has developed again. After that, 200J of defibrillation was performed 6 times, but VF continued. A pair of defibrillation patches were attached to the front and back of the chest additionally (Fig. 1A,1B). After 4 times of DSD, VF was terminated. After ROSC, ECG rhythm was accelerated idioventricular rhythm and vital signs were stable (Fig. 2 A). The patient was admitted to the intensive care unit (ICU). On the eighth hospital day, he was discharged to the nursing hospital. The Cerebral Performance Category (CPC) Scale was 4.

### 2.2 Case 2

A 35-year-old man without any medical history had a seizure while taking a shower and was reported to the paramedics. Recognizing the cardiac arrest, the dispatcher instructed dispatch-assisted CPR and the witness started CPR. The initial ECG rhythm at the scene was VF, and a total of 4 defibrillations was performed with the energy of 150 J until arriving at the ED but the spontaneous circulation wasn’t recovered. The initial rhythm at the ED was VF, so 200J of single defibrillation was tried. The Advanced Cardiac Life Support was performed according to the guidelines, but the VF lasted for 10 minutes. After the DSD of 300J once and 400J twice, ROSC was achieved (Fig. 1A,1B). After ROSC, ECG showed a junctional rhythm with a descent of the ST segment and vital signs were stable (Fig. 2 -B). The patient was admitted to the ICU. On the 13th hospital day, he was discharged to the nursing hospital. The CPC Scale was 4.

### 2.3 Case 3

A 38-year-old man with hypertension visited the ED by an ambulance. He had chest pain that started 30 minutes ago. He was unconscious shortly after arriving in the ED, and VF was observed on the ECG. Despite repeated defibrillation of 200J and adherence to Advanced Cardiac Life Support, VF was sustained. After the 3 times of 400J DSD, he recovered a spontaneous circulation (Fig. 1A,1B). ECG rhythm was arrhythmia including early ventricular contraction. Vital signs were stable (Fig. 2 -C). After emergency coronary angiography, he was admitted to the ICU. Coronary arteriography findings were total occlusion of a left anterior descending artery and 80% tubular occlusion of a right coronary artery. He was transferred to the general ward 2 days later. On the 8th day, he was discharged home with the CPC Scale 1.

## 3 Discussion

The origins of CPR start with studies by Kouwenhoven et al. in 1960.^[6]^ Since then, the American Heart Association (AHA) and the American Red Cross have begun to apply the guidelines for CPR in 1966. Korea has also been developing and distributing guidelines based on the Korean situation since the establishment of the Korean Association for Cardiopulmonary Resuscitation in 2002. The AHA provides updated guidelines every five years to raise the likelihood of rejuvenation and presents a series of courses as five levels of chains of survival.^[7,8]^ If the ECG rhythm of the collapsed victim was VF or pulseless VT, the probability of survival is reduced by about 10% for every 1 minute of defibrillation delay.^[9,10]^ Early defibrillation is the most fundamental therapy in lethal arrhythmia. In this regard, the importance of early defibrillation in the prehospital stage should be emphasized.^[11–13]^ The definition of RVF is that the ECG rhythm is not recovered even after three or more single defibrillation and appropriate medication. There are several reported cases that DSD applied to RVF patients.^[14,15]^ In general, the defibrillation electrodes can apply to the anterior-posterior attachment method (with the left ventricle / between the two scapulas) or the external attachment method (Bust of the sternum / Lower left of the pectoral muscle).^[16]^ DSD is a method of applying two pair of defibrillators to a patient and simultaneously delivering a dual shock. Apply one set of pads to the front-back and another set of pads to the front-side.^[17]^ It is important to make sure that the two pads on the front side do not contact each other. Charge the monophasic defibrillator to 360J and the biphasic defibrillator to 200J. Because double energy is applied at the same time, it gives 720J for a monophasic and 400J for a biphasic defibrillator. Although defibrillation failure has been reported in the RVF despite the application of DSD, successful defibrillation is achieved in most cases using DSD. In each of our cases, single defibrillation of 150J was performed 14 times, 5 times, and 3 times at the scene, respectively. But VF was sustained. After arriving at ED, 4 times, 3 times, and 3 times of DSD attempted, then ROSC was achieved. The reason why the CPC score of a case 1 and 2 patient is not as good as that of case 3 is that the patient in case 3 was collapsed just after arrival at the ED and was immediately recognized by the medical team. It is also believed that the fast application of the DSD within 10 minutes affected the patient's prognosis. In Korea, paramedics are allowed by law to perform limited prehospital care just as much as Emergency Medical Technician-Intermediate (EMT-I) in the United States. They cannot use antiarrhythmics such as amiodarone. Therefore, if they meet RVF patients at the scene, the only resource they can use is defibrillation. As is well known, if the ECG rhythm of the cardiac arrest patient shows VF or pulseless VT, delayed application of defibrillation reduces the chance of survival. Currently, the application of DSD is not a standard therapy, but it can be tried as a reasonable option for patients without response to conventional therapy for RVF. The three cases we have shown are small, but DSD improves the chance of spontaneous circulation. Therefore it is suggested that attempts of DSD to patients with RVF, especially in the prehospital stages as a way to improve the ROSC.^[18]^ The difference between the existing studies is that although the case of this study did not lead to high scores of CPC, the existing study had a high percentage of deaths, but it survived all three cases and enabled nursing or daily life. This work is a case-reporting study to present the effectiveness of Double Sequential Defibrillation (DSD), which requires well-defined studies such as RCT in the future.(Class 2b, LOE C-LD, AHA Guideline 2020).^[19]^

## Author contributions

**Conceptualization:** Hyun Noh.

**Data curation:** Hyo Jeong Choi.

**Formal analysis:** Hyo Jeong Choi.

**Investigation:** Hyo Jeong Choi.

**Methodology:** Hyo Jeong Choi.

**Writing – original draft:** Hyo Jeong Choi.

**Writing – review & editing:** Hyun Noh.

## References

[1] Rea TD Eisenberg MS Sinibaldi G. Incidence of EMS-treated out-of-hospital cardiac arrest in the United States. Resuscitation 2004;63:17–24. 15451582 10.1016/j.resuscitation.2004.03.025

[2] Vaillancourt C Stiell IG. Cardiac arrest care and emergency medical services in Canada. Can J Cardiol 2004;20:1081–90. 15457303

[3] Cobb LA Fahrenbruch CE Olsufka M. Changing incidence of out-of hospital ventricular fibrillation, 1980-2000. JAMA 2002;288:3008–13. 12479765 10.1001/jama.288.23.3008

[4] Kudenchuk PJ Cobb LA Copass MK. Amiodarone for resuscitation after out-of-hospital cardiac arrest due to ventricular fibrillation. N Engl J Med 1999;341:871–8. 10486418 10.1056/NEJM199909163411203

[5] Leyda Hu DO Winny Liang PA-C Richard Cousino DO. Double Sequential Defibrillation for Refractory Ventricular Fibrillation and Pulseless Ventricular Tachycardia. 2017. Emergency Medicine 2017;49:499–504.

[6] Kouwenhoven WB Jude JR Knickerbocker GG. Closed chest cardiac massage. JAMA 1960;173:1064–7. 14411374 10.1001/jama.1960.03020280004002

[7] Lee MH Kim MR Eom YJ. The usefulness of HFABP and IMA to diagnose the cardiac cause of arrest and the difference of the two biomarkers between the ROSC group and the non-ROSC group. J Korean Sco Emerg Med 2009;20:50–7.

[8] Travers AH Rea TD Bobrow BJ. part 4. CPR overview: 2010 American Heart Association guidelines for cardiopulmonary resuscitation and emergency cardiovascular care. Circulation 2010;122:S676–84. 20956220 10.1161/CIRCULATIONAHA.110.970913

[9] Eisenberg MS Horwood BT Cummins RO. Cardiac arrest and resuscitation; a tale of 29 cities. Ann Emerg Med 1990;19:179–86. 2301797 10.1016/s0196-0644(05)81805-0

[10] Larsen MP Eisenberg MS Cummins RO. Predicting survival from out-of hospital cardiac arrest: a graphic model. Ann Emerg Med 1993;22:1652–8. 8214853 10.1016/s0196-0644(05)81302-2

[11] Kinney KG Boyd SY Simpson DE. Guidelines for appropriate in-hospital emergency team time management: the Brooke Army Medical Center approach. Resuscitation 2004;60:33–8. 14987781 10.1016/S0300-9572(03)00259-4

[12] Spearpoint KG McLean CP Zideman DA. Early defibrillation and the chain of survivial in ‘in-hospital’ adult cardiac arrest; minutes count. Resuscitation 2000;44:165–9. 10825615 10.1016/s0300-9572(00)00158-1

[13] Bossaert L. Automated External Defibrillation. European Resuscitation Council Guidelines For Resuscitation. Amsterdam: Elsevier, 1998:217.

[14] Lybeck AM Moy HP Tan DK. Double sequential defibrillation for refractory ventricular fibrillation: a case report. 2015. Prehosp Emerg Care 2015;19:554–7. 25970000 10.3109/10903127.2015.1025155

[15] Johnston M Cheskes S Ross G. Double sequential external defibrillation and survival from out-of-hospital cardiac arrest: a case report. Prehosp Emerg Care 2016;20:662–6. 27077941 10.3109/10903127.2016.1168891

[16] Hyeong yeong Park. Tips for Defivrillation/Cardioversion. Pacing, Sao_2_/EtCO_2_ monitoring. Ann Emerg Med 2015;2:329–32.

[17] Merlin MA Tagore A Bauter R. A case series of double sequence defibrillation. Prehosp Emerg Care 2016;20:550–3. 26848018 10.3109/10903127.2015.1128026

[18] Cortez E Krebs W Davis J. Use of double sequential external defibrillation for refractory ventricular fibrillation during out-of-hospital cardiac arrest. Prehosp Emerg Care 2016;108:82–6. 10.1016/j.resuscitation.2016.08.002 27521470

[19] Panchal AR Bartos JA Cabañas JG. Part 3: adult basic and advanced life support 2020 AHA guidelines for CPR and ECC. Circulation 2020;142: supple 2: S366–468. 33081529 10.1161/CIR.0000000000000916

